# Tuning the Functional Groups on Carbon Nanodots and Antioxidant Studies

**DOI:** 10.3390/molecules24010152

**Published:** 2019-01-02

**Authors:** Zuowei Ji, Alex Sheardy, Zheng Zeng, Wendi Zhang, Harish Chevva, Kokougan Allado, Ziyu Yin, Jianjun Wei

**Affiliations:** Department of Nanoscience, Joint School of Nanoscience and Nanoengineering, University of North Carolina at Greensboro, Greensboro, NC 27401, USA; z_ji@uncg.edu (Z.J.); atsheard@uncg.edu (A.S.); hdzengzheng@163.com (Z.Z.); w_zhang3@uncg.edu (W.Z.); h_chevva@uncg.edu (H.C.); k_allado@uncg.edu (K.A.); z_yin@uncg.edu (Z.Y.)

**Keywords:** carbon nanodots, functional groups, antioxidation, radical scavenging, electro-chemistry, charge transfer

## Abstract

Carbon nanodots (CNDs) have shown good antioxidant capabilities by scavenging oxidant free radicals such as diphenyl-1-picrylhydrazyl radical (DPPH•) and reactive oxygen species. While some studies suggest that the antioxidation activities associate to the proton donor role of surface active groups like carboxyl groups (–COOH), it is unclear how exactly the extent of oxidant scavenging potential and its related mechanisms are influenced by functional groups on CNDs’ surfaces. In this work, carboxyl and the amino functional groups on CNDs’ surfaces are modified to investigate the individual influence of intermolecular interactions with DPPH• free radical by UV-Vis spectroscopy and electrochemistry. The results suggest that both the carboxyl and the amino groups contribute to the antioxidation activity of CNDs through either a direct or indirect hydrogen atom transfer reaction with DPPH•.

## 1. Introduction

Carbon nanodots (CNDs) have been explored for applications in biomedicine, sensors, optoelectronics and catalysis [1,2,3,4], owing to discovered superior biocompatibility and outstanding optoelectronic properties [5,6]. Various synthetic methods used for the preparation of CNDs have been classified into “top-down” route and “bottom-up” route [7]. The “top-down” route includes chemical ablation [8,9], electrochemical carbonization [10] and laser ablation [11,12]; and “bottom-up” route has hydrothermal/solvothermal treatment [13,14], and microwave irradiation [15,16]. Amongst these, the rapid, cost effective, scalable and eco-friendly features have made the microwave irradiation synthesis very popular these days [17].

Recently, CNDs were reported as good antioxidant agents in free radical scavenging [18]. It is known that free radicals generated from the metabolism of oxygen in living systems are very chemically reactive due to their unpaired valence electrons [19]. The damage caused by free radicals, such as lipid peroxidation, DNA damage, and protein oxidation, will accelerate the ageing process [19]. CNDs’ properties as electron donors or electron acceptors endow them with potential applications in antioxidants or prooxidants [20], depending on the activities of the unpaired valence electrons. Reportedly, their role of antioxidant has been attested by a series of nitrogen-centered 2-2-diphenyl-1-picrylhydrazyl radical (DPPH•) based assay [21,22]. Researchers have proposed that the antioxidation of CNDs is mainly due to the function of surface functional groups of CNDs, such as carboxyl groups that could act as proton donors which convert DPPH• into a stable DPPH-H complex through a HAT mechanism [23,24]. However, the evidence of the direct involvement of carboxyl groups in the antioxidation of CNDs is still absent. Furthermore, the electron transfer from the aromatic primary amines to the hydrazyl in organic chemistry suggested by Hammond, et al [25], leads to a hypothesis that the primary amines existing on the surface of CNDs also participate in the antioxidation process.

Understanding the antioxidation reactions in radical scavenging would benefit the design of nanomaterials-based agents in therapies and treatment in the field of oxidative stress. In literature, antioxidation reactions of many types of nanoparticles (NPs) have been documented and the antioxidant mechanisms are reported with respects to the compositions of the particles or surface functionalization [26,27]. For example, gold nanoparticles (AuNPs) functionalized with Trolox, a water-soluble vitamin E analogue, demonstrated enhanced DPPH• scavenging activity involving a HAT process [28]. The antioxidant activity engaged the capacity of reducing gold (III) to gold particles [27]. The catalase-mimic activity of cerium oxide NPs and Co_3_O_4_ NPs has been reported as antioxidants for superoxide scavenging by a direct charge exchange reaction at the surface of the NPs [29,30]. More recently, γ-Al-NPs was reported to have antioxidant activities towards scavenging DPPH• free radicals involving the surface adsorption followed by interactions with the acidic and basic sites at the surface of γ-Al-NPs [31].

In order to test the hypothesis regarding functional groups, specifically the carboxyl groups (–COOH) and amino groups(–NH_2_) of CNDs in DPPH• radical scavenging, an idea was proposed to explore the roles of –COOH and –NH_2_, respectively, in the process of antioxidation by blocking one of them using organic chemistry methodology. Reportedly, EDC/NHS coupling has been utilized to conjugate the polymeric nanoparticles with the specific antibody by converting the carboxyl groups to the NHS-esters [32]. Researchers have also reported the blocking of amino groups of proteins by citraconic anhydride [33]. Thus, citraconic anhydride was tried to block the primary amines of CNDs for the first time in this work.

We report herein microwave irradiation assisted synthesis of CNDs using glucosamine hydrochloride as the only precursor [34]. Further, carboxyl groups and amino groups on the CNDs were blocked by a modified EDC/NHS reaction and citraconic anhydride method, respectively. The prepared CNDs were characterized with various spectroscopic and microscopic techniques. Lastly, the antioxidation capability of the CNDs before and after blocking was evaluated by UV-Vis spectroscopic and electrochemical assays.

## 2. Results and Discussion

### 2.1. CNDs Synthesis and Characterization

**Figure 1:**
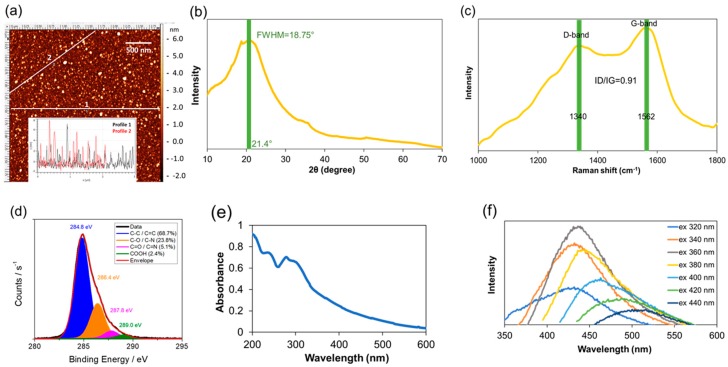
Characterization of unmodified CNDs. (**a**) AFM imaging with representative z-axis profile (inserted); (**b**) XRD spectrum; (**c**) Raman spectrum; and (**d**) XPS spectra of the CNDs; (**e**) UV−vis absorption spectrum; (**f**) Fluorescence emission spectra of the CNDs in deionized water obtained at different excitation wavelengths with a 20 nm increment from 320 to 440 nm.

In this work, a green and facile microwave synthesis was employed to generate fluorescent CNDs using glucosamine hydrochloride as the only precursor. In order to visualize the size distribution of CNDs and the size changes after function group modification, we selected AFM imaging to obtain size distribution of CNDs, because the z-axis in AFM is accurate with angstrom resolution. The analysis from the AFM images (Figure 1a and Figure S1) reveals the size distribution of the unmodified CNDs of an average 2.0 ± 0.7 nm. After modifications, the size of the –COOH-blocked (Figure S1a) and –NH_2_-blocked (Figure S1b) CNDs increases to average around 3 nm and 4 nm, respectively. This is due to additional structures attached to the edge of CNDs. The unmodified CNDs were characterized with a few spectroscopic techniques. XRD of the CNDs (Figure 1b) shows a diffraction peak at 21.4° with a full width at half maximum (FWHM) of 18.75°, indicating an interlayer distance between the planar carbon-based sheets for disordered graphite structure is about 0.41 nm [35]. Figure 1c shows the Raman spectrum with D bands at 1340 cm^−1^ (sp^3^-hybridized) and G bands at 1562 cm^−1^ (sp^2^-hybridized), which presents an intensity ratio *I_D_/I_G_* of 0.91 [36]. The carbon 1s XPS spectrum (Figure 1d) shows the presence of the functionalities at the surface, like C–C and C=C (68.7%, 284.8 eV), C–O and C–N (23.8%, 286.4 eV), C=O and C=N (5.1%, 287.8 eV), and COOH (2.4%, 289.0 eV) [37]. The UV-Vis spectrum (Figure 1e) exhibits two features in the UV region (200–400 nm) with a tail extending to the visible range. The first feature is an absorbance peak at 240 nm, attributed to π-π* transitions of the aromatic C–C bonds, and the second feature is a shoulder at 290−300 nm, assigned to the n-π* transition of the C=O bonds [38]. The fluorescence emission spectra of CNDs in deionized water at various excitation wavelengths ranging from 320 nm to 440 nm is depicted in Figure 1f. The maximum peak of fluorescence emission at 435 nm was observed at an excitation wavelength of 360 nm. In addition, the fluorescence spectra display a red-shift pattern with increasing excitation wavelength. These results suggest that the unmodified CNDs have a spherical morphology, and structurally possess a graphitic core with surface functional groups such as carboxyl and amines/amides.

### 2.2. Surface Modification of CNDs

After a variety of characterization of the unmodified CNDs, the blocking experiments of the carboxyl groups and amino groups were carried out separately. It is known that 1-(3-dimethyl-aminopropyl)-3-ethylcarbodiimide hydrochloride (EDC, TCI) is a zero-length crosslinking agent used to couple carboxyl groups to primary amines [39]. Due to the interaction of the carboxyl group with carbodiimide carbon of EDC, an unstable *O*-acylurea adduct is formed [40]. In order to increase the stability of this active ester and the efficiency of EDC-mediated coupling reactions, *N*-hydroxysuccinimide (NHS) was added to this reaction. The presence of NHS, along with EDC, is thought to form an amine-reactive NHS-ester that is considerably more stable and productive than the *O*-acylurea intermediate [41,42]. Following an adapted protocol [37], the blockage of the carboxyl groups on the CNDs was achieved by incubating the CNDs suspension with 0.5 mM EDC/NHS. The –COOH-blocked CNDs shows good distribution and a slightly increased size (Figure S1a).

Previous studies have demonstrated that the addition of citraconic anhydride to primary amines results in the citraconic amide formation. Additionally, the primary amine functionality could be regenerated again by the hydrolyzation of the citraconic amides under acidic conditions [43]. This reaction proceeds through a nucleophilic substitution mechanism. Briefly, the electron pair from the nucleophile(–NH_2_) attacks the carbonyl carbon of the citraconic anhydride, forming a new bond namely the amide bond [44]. Because of the asymmetry of the citraconic anhydride structure, two constitutional isomers are possible to form depending on which carbonyl carbon of citraconic anhydride is attacked [33,44]. The relatively large size of CNDs (Figure S1b as compared to small organic molecules) is likely due to steric interference.

### 2.3. Characterization of the Functional Group Blocked CNDs

**Figure 2:**
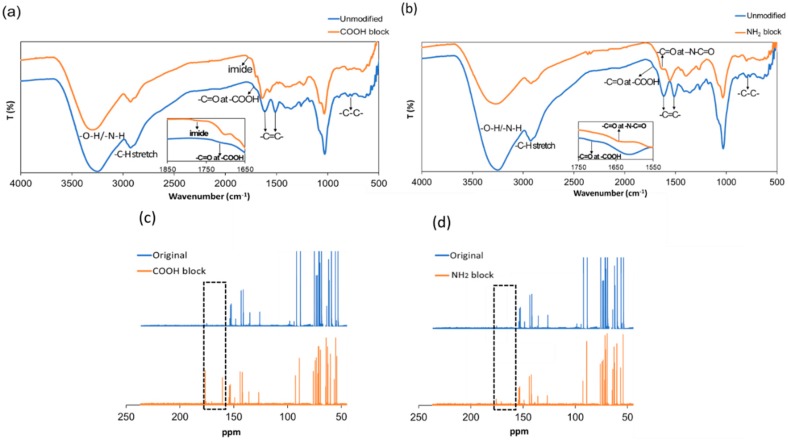
FTIR spectra comparison of the unmodified CNDs, –COOH-blocked CNDs (**a**), and –NH_2_-blocked CNDs (**b**) and ^13^C NMR spectra comparison of the unmodified CNDs, –COOH-blocked CNDs (**c**), and –NH_2_-blocked CNDs (**d**). Each NMR spectrum is the average of 10,000 scans.

Elemental analysis was measured by energy-dispersive X-ray (EDX) spectroscopy. Figures S2–S4 present the percentage of carbon, hydrogen, oxygen, and nitrogen of unmodified, –COOH-blocked, –NH_2_-blocked CNDs, respectively. As expected, N and O atom percentage increases from 1.36% to 1.83% (the N/C ratio from 0.1370 to 0.1560) and 5.63% to 5.74% (O/C atom ratio from 0.5675 to 0.4893) in –COOH-blocked CNDs as a comparison to unmodified CNDs due to the addition of NHS-ester groups. For –NH_2_-blocked CNDs, the addition of citraconic anhydride increases the C and O elements, which should decrease the N/C atom ratio in –NH_2_-blocked CNDs as a comparison to unmodified CNDs, resulting in 0.1294 from 0.1370 in the EDX measurement. The chemical structures of the unmodified, –COOH-blocked, and –NH_2_-blocked CNDs were characterized by FTIR spectroscopy (Figure 2a,b). The broad bands at 3100–3400 cm^−1^ in all CND samples are assigned to O–H and N–H-functionalities, accounting for the solubility and stability of the CNDs in aqueous media. The IR transitions at 1512 cm^−1^ and 1613 cm^−1^ are assigned to aromatic C=C vibrations [45]. The bands around 1710 cm^−1^ are attributed to the C=O vibrations [45]. However, the characteristic carboxyl stretching vibrations of simple ketones and carboxylic acids around 1710 cm^−1^ observed in unmodified CNDs shifts to that of imides at 1776 cm^−1^ after the carboxyl groups were blocked (Figure 2a) [46]. The presence of the IR transitions at 1567 cm^−1^ of –COOH-blocked CNDs was corresponding to the aromatic C=C vibrations [46]. In the case of –NH_2_-blocked CNDs, the IR transitions displayed at 1552 cm^−1^ and 1633 cm^−1^ were attributed to aromatic C=C vibrations and C=O of amides, respectively (Figure 2b) [46]. A comparison of ^13^C NMR spectra of the unmodified, –COOH-blocked and –NH_2_-blocked CNDs give additional evidence for the blocking reaction (Figure 2c,d). In the case of the unmodified CNDs, the characteristic signal of carboxylic acid was at about 177 ppm (Figure 2c) [46]. The intensity of the carboxylic acid is not that strong as shown here, which is in agreement with our XPS result that the ratio of carboxylic acid was about 2.4% in unmodified CNDs (Figure 1d). The new peaks appeared at 161, 171 and 179 ppm representing the presence of esters were formed in the –COOH-blocked CNDs (Figure 2c) [46]. The similar shift of the carbon resonance to lower field at about 172 ppm corresponding to the amide bond was observed in the case of –NH_2_-blocked CNDs (Figure 2d) [46]. With all the results taken together, it is obvious that the blocking process has taken place at the surface of CNDs successfully.

### 2.4. UV-Vis Study of Antioxidation Activity

The antioxidant activity measurement is used to evaluate the capability of a material to scavenge or neutralize free radicals. Particularly, DPPH• is considered as one of the best analytes for assessing the antioxidation activity owing to its good stability, high catalytic activity, and ease of handling. Herein, a DPPH• reduction assay was employed to investigate the antioxidation activity of CNDs via UV-Vis spectroscopy. The free radical in DPPH• is stable due to resonance delocalization of the spare electron and has absorption at 517 nm in methanol solution [47]. Upon accepting a hydrogen radical from an antioxidant, the conjugated π system is shrunk, resulting in a decrease in 517 nm absorption and color change from deep violet to light yellow [22]. Therefore, the unreacted DPPH• could be quantified by comparing absorbance at 517 nm against the blank, and the antioxidation activity can be calculated according to the following equation:

(1) $$Antioxidation\cdot activity=\frac{A_{0}-A_{C}}{A_{0}}\times 100\%$$

where *A*_0_ and *A_C_* are corresponding to the absorbance of DPPH• at 517 nm in the absence and presence of CNDs, respectively.

**Figure 3:**
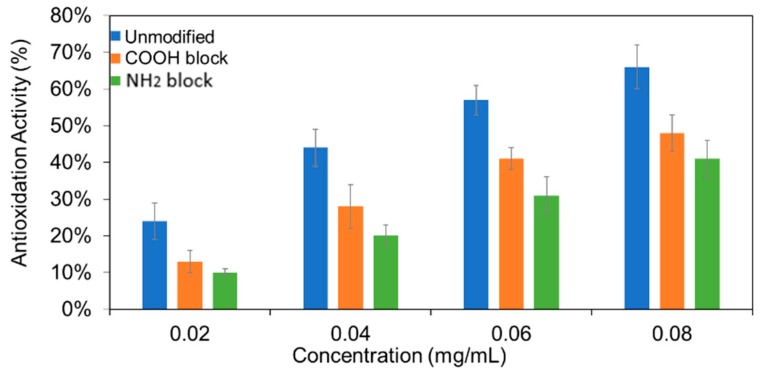
The calculated antioxidation activity of unmodified, –COOH-blocked, and –NH_2_-blocked CNDs for multiple concentrations. Each measurement at a given concentration was repeated three times. The means and standard deviations were calculated.

Representative absorption spectra of 0.02 mg/mL DPPH• methanol solution with different concentrations of unmodified, –COOH-blocked, and –NH_2_-blocked CNDs measured after reaction for 1.5 h in the dark was shown in Figure S5. The final, averaged antioxidant activities are presented in Figure 3. The antioxidation activities of the unmodified CNDs were calculated to be 24%, 44%, 57%, and 66% for concentrations of 0.02, 0.04, 0.06, and 0.08 mg/mL, respectively. For the –COOH-blocked CNDs, the antioxidation activities decreased 45%, 36%, 28%, and 27% for concentrations of 0.02, 0.04, 0.06, and 0.08 mg/mL, respectively, compared to the unmodified CNDs. Meanwhile, 58%, 54%, 46%, and 38% decreases corresponding to concentration of 0.02, 0.04, 0.06, and 0.08 mg/mL were observed in the case of –NH_2_-blocked CNDs. A minimum three trials of each concentration were conducted. EDC/NHS and citraconic anhydride in DPPH• were conducted as controls (Figure S6).

### 2.5. Electrochemical Study of DPPH• Scavenging Activity

**Figure 4:**
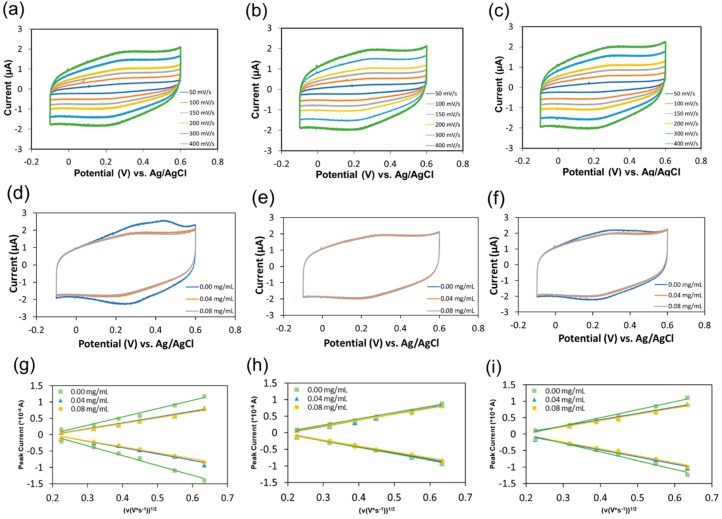
Representative cyclic voltammograms for the DPPH•-gold electrode system reacted with 0.04 mg/mL unmodified (**a**), –COOH-blocked (**b**), and –NH_2_-blocked CNDs (**c**) at scan rates of 50, 100, 150, 200, 300 and 400 mV/s, respectively; representative comparison of cyclic voltammograms for the DPPH•-gold electrode system reacted with different concentrations of the unmodified (**d**), –COOH-blocked (**e**), and –NH_2_-blocked CNDs (**f**) at a scan rate of 400 mV/s; representative plots of the linear dependence of the peak current on the voltage scan rate reacted with different concentrations of the unmodified (**g**), –COOH-blocked (**h**), and –NH_2_-blocked CNDs (**i**).

An electrochemical platform established in our group was also employed to evaluate the antioxidant capacity of the three types of CNDs [23]. A pair of redox peaks were observed from the cyclic voltammograms at −0.1–0.6 V voltage window of DPPH• (0.02 mg/mL) reacted with different types of CNDs (0.04 mg/mL) at different scan rates (Figure 4a–c). The representative cyclic voltammograms for the DPPH•-gold electrode system reacted with three types of CNDs at different concentrations were shown in Figure S7. Additionally, a dramatic decrease in the intensity of the anodic and cathodic peaks was found (Figure 4d) with an increment of the concentration of the unmodified CNDs. However, this tendency was not as obvious in the case of the two types of blocked CNDs (Figure 4e,f). By comparing the intensity of the anodic and cathodic peaks resulting from the electrochemical oxidation and reduction of DPPH•, respectively, the antioxidant capacity of the three types of CNDs was determined.

**Figure 5:**
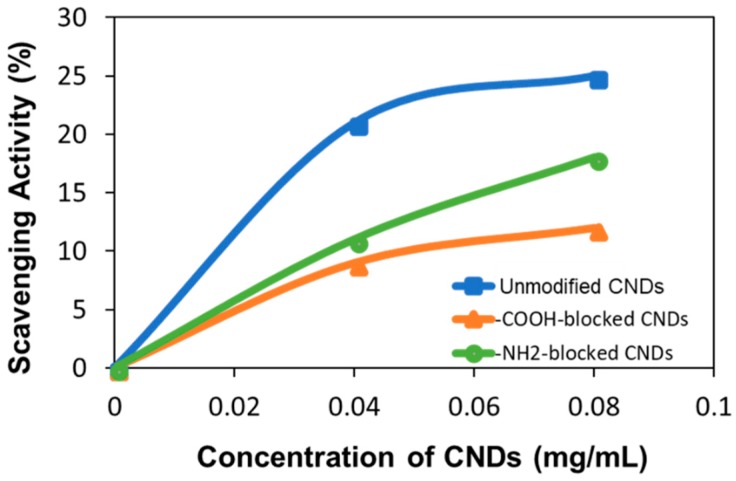
The tendency of the averaged DPPH• scavenging activity of the three types of CNDs as a function of concentration. The standard deviation of the data points is listed in Table 1.

A linear dependence of the peak current, *ip* (A), with the square root of their corresponding voltage scan rate at different concentrations of the unmodified (Figure 4g), –COOH-blocked (Figure 4h), and –NH_2_-blocked CNDs (Figure 4i) was obtained. This linear dependence can then be used to calculate the diffusion coefficient (*D*_0_, cm^2^/s) of DPPH• without CNDs via Randles-Sevcik equation [48,49]:

(2) $$ip=\left(2.69\times 10^{5}\right)\;n^{3/2}ACD_{0}^{1/2}v^{1/2}$$

where *n* is the number of electrons exchanged, *A* is the active surface area of the gold electrode (0.043 cm^2^), *C* is the concentration of the DPPH• (mol/mL), *ν* is the voltage scan rate (V/s). Using the same equation and the calculated diffusion coefficient, the concentration of unreacted DPPH• after CNDs treatment can then be calculated for each type and concentration of CNDs (Table S1). The scavenging activity can be calculated using Equation (3), where *C*_0_ and *Cc* are the concentration of DPPH• in the absence and presence of CNDs, respectively. Applying Equation (3) gives scavenging activities of the unmodified, –COOH-blocked CNDs, and –NH_2_-blocked CNDs which were obtained to be 21%, 9%, and 11% at concentration of 0.04 mg/mL and 25%, 12%, and 18% at concentration of 0.08 mg/mL (Figure 5). The standard deviations are listed in Table 1.

(3) $$Scavenging\cdot activity=\frac{C_{0}-C_{C}}{C_{0}}\times 100\%$$

Comparing the antioxidation activity results of electrochemical assay and UV-Vis assay, it is interesting to find that, in UV-vis assay, the NH_2_-blocked CNDs show less antioxidation activity than that of –COOH-blocked CNDs, and the electrochemical assay presents opposite way. While elemental and structural analysis convinced the success of surface functional group modification, quantitative –COOH and –NH_2_ groups in the CNDs are not able to obtain. It is unclear which functional group is more effective in the antioxidation. Nevertheless, these results indicate both -COOH and –NH_2_ contribute significantly for the antioxidative activity.

**Scheme 1:**
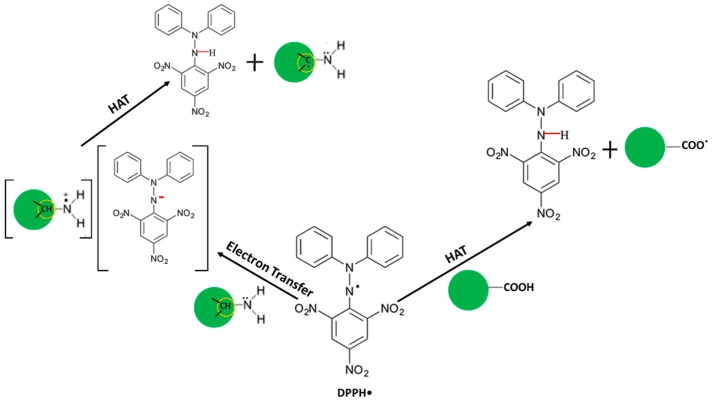
Schematic description of the DPPH• based antioxidation mechanism of CNDs.

Taking all the results together, we have proved that both carboxyl groups and amino groups participate in the antioxidation process of CNDs based on a DPPH• reduction assay. It was reported that DPPH• can be quenched by hydrogen atom transfer [50], electron transfer [51] or a combination of the two [52]. In the case of CNDs, the proposed mechanisms for DPPH• scavenging are described in Scheme 1. On one hand, DPPH• is converted into DPPH-H complex by taking up a hydrogen donated by the carboxyl groups through a direct hydrogen atom transfer (HAT) mechanism [23,24]. On the other hand, we proposed here an indirect HAT mechanism (the single electron transfer followed by proton transfer) to explain the participation of amino groups in the antioxidation of CNDs. Concretely, a fast electron transfer from the primary amines to the DPPH• could be justified by the electron-donor character of the –NH_2_ group, forming a complex of an aminium cation radical and DPPH anion [51,53]. After the electron transfer, the aminium cation radical undergoes deprotonation to give DPPH-H complex [53]. Both the step of electron transfer and deprotonation are favored by the polarity of the methanol solution which promotes the necessary solvation to stabilize the ionic species [54]. The mechanism raised here in turn explains our UV-Vis and electrochemical results well. Therefore, we believe that both the carboxyl groups and the amino groups contribute to the antioxidation activity of the CNDs through either a direct or indirect HAT mechanism.

Based on the HAT mechanism, the de/protonation of –COOH and –NH_2_ groups would reflect their antioxidative activities. The protonation of these groups is predicted to increase the DPPH• radical scavenging. To test this, the UV-vis assay of DPPH• at three different pH values (3, 7, 11) were performed using 0.02 mg/mL concentration of unmodified CNDs. The results (Figure S8) demonstrate that the antioxidative activity increases in acidic solution and decrease in basic solution. The –COOH-blocked or –NH_2_-blocked CNDs also present the similar tendency as the unmodified CNDs. This proves that protonation of carboxyl and amine groups in the acidic solution provides more hydrogen atoms in the antioxidation process and thus more effective in radical scavenging.

## 3. Experimental

### 3.1. Synthesis of CNDs

The synthesis of CNDs was performed using glucosamine hydrochloride (Alfa Aesar, Haverhill, MA, USA) as a precursor in a microwave synthesizer (908005 Microwave Reactor Discovery System, CEM Corp, Matthews, NC, USA). Briefly, glucosamine hydrochloride (0.1 g) was dissolved completely in deionized water (1.0 mL) and then heated in the microwave reactor for 9 minutes at a power of 300 W and at temperature of 150 °C. Afterwards, the aqueous reaction mixture was purified using a centrifuge at 3500 rpm for 5 min to separate the large aggregated particles from the dark-brown supernatant. The homogeneous solution was then dialyzed (MWCO 1000 Da, Aldrich, St. Louis, MO, USA) against DI water for 24 h. Finally, the purified solution was dried using a freeze dryer for 36 h to obtain the solid sample.

### 3.2. Characterization of CNDs

Atomic force microscopy (5600LS AFM, Agilent Technologies, Tempe, AZ, USA) imaging on fresh mica surfaces was used to obtain the size distribution of CNDs. Elemental composition and chemical structure of the CNDs was assessed by Energy-dispersive X-ray (EDX) spectroscopy analysis using the S-4800-I FESEM w/Backscattered Detector (Carl Zeiss NTS GmbH, Oberkochen, Germany), Fourier transform infrared spectroscopy (FTIR, Varian 670, Agilent Technologies, Santa Clara, CA, USA), Raman spectroscopy (XploRA One Raman Confocal System, Horiba, Villeneuve, France), carbon 1s X-ray photoelectron spectroscopy (XPS, ESCALAB 250 Xi, Thermo Fisher, West Sussex, UK), and X-ray powder diffraction (XRD, Agilent Technologies Oxford Gemini, The Woodlands, TX, USA). Solid samples were redispersed in D_2_O for ^13^C NMR (Agilent 700 MHz NMR, Agilent Technologies, Santa Clara, CA, USA) analysis. Optical properties were measured using ultraviolet-visible spectroscopy (UV-Vis spectroscopy, Cary 6000i, Varian, Agilent Technologies, Santa Clara, CA, USA) and fluorescence spectroscopy (Fluoro-Max 4 fluorometer, Horiba, Kyoto, Japan), respectively.

### 3.3. Preparation of the Blocked CNDs

In the case of blocking the carboxyl groups on the surface of CNDs, the CNDs were first re-dispersed in deionized water with a final concentration of 0.3 mg mL^−1^. The homogeneous solution was then reacted with a solution containing 0.5 mM 1-(3-dimethylaminopropyl)-3-ethylcarbodiimide hydrochloride (EDC, TCI)/0.5 mM *N*-hydroxysuccinimide (NHS, Aldrich) for 2 h to block the carboxyl groups. To block the amino groups on the surface of the CNDs, the CNDs with a final concentration of 0.2 mg mL^−1^ was treated with citraconic anhydride (Aldrich, 0.03 mg mL^−1^) and the pH was adjusted to 6.0 by adding 5N-NaOH. The system was kept at room temperature with stirring for 2 h. For both carboxylic acid- and amine-blocked CNDs, unreacted reagents were removed by a 24 h dialysis process (MWCO 1000 Da, Aldrich) against DI water. Both samples were then frozen and freeze dried for 36 h to obtain solid powders.

### 3.4. UV-Vis study of Antioxidation Activity

The antioxidation activity of the blocked CNDs was compared to the unmodified CNDs using the DPPH• (Alfa Aesar)-based assay according to previously reported procedure [23]. A series of CND concentrations ranging from 0 to 0.08 mg/mL were mixed with DPPH• at a final concentration 0.02 mg/mL in absolute methanol (Fisher Scientific, Waltham, MA, USA). The reaction was kept in a dark environment for 1.5 h, and then the absorbance at 517 nm was measured via UV-Vis spectroscopy. Each sample was conducted for at least three trials.

### 3.5. Electrochemistry Study

The electrochemical experiments were performed with a three-electrode electrochemical cell containing a gold working electrode (Fisher Scientific), an Ag/AgCl reference electrode (Fisher Scientific), and a platinum counter electrode (Fisher Scientific). For all experiments, 10.0 mL of a 1:1 mixture of absolute methanol and phosphate buffer solution (Life Tech, Carlsbad, CA, USA) containing 0.02 mg/mL (52 nmol/mL) DPPH• reacted with multiple concentrations of one type of CNDs. Each system was maintained in a dark environment for 45 mins reaction. Cyclic voltammetry of each of the samples was performed at room temperature with six different scan rates. A solution of 0.02 mg/mL of DPPH• in 10.0 mL methanolic phosphate buffer without CNDs was used as a control.

## 4. Conclusions

The routes to manipulation of the carboxyl groups and amino groups on the CNDs provide an extraordinary opportunity to study the roles of the functionalities of CNDs in terms of their antioxidant capabilities, and potentially for other properties. Apart from this, the antioxidant results of the modified CNDs suggest that not only the carboxyl groups play an important role in antioxidation through a HAT mechanism, but also the amino groups via an electron transfer coupled indirect HAT mechanism. This work would promote a better understanding of this type of complicated nanomaterials and help the design and tuning structures of the CNDs, and thus facilitate the development of its practical application in the field of biomedicine.

## Figures and Tables

**Table 1:** The standard deviations of the averaged DPPH• scavenging activity of the three types of CNDs with different concentrations.

Concentration of CNDs (mg/mL)	Unmodified CNDs (%)	–COOH-Blocked CNDs (%)	–NH_2_-Blocked CNDs (%)
0.04	±14	±7	±9
0.08	±14	±4	±4

## Supplementary Materials

Additional supporting data of CND characterization, UV-vis spectra, control experiments, and electrochemical characterization. Figure S1. AFM topography image of –COOH-blocked (a) and –NH_2_-blocked CNDs (b); and the representative AFM height profiles. Figure S2. EDX elemental analysis of un-modified CNDs. Figure S3. EDX elemental analysis of –COOH-blocked CNDs. Figure S4. EDX elemental analysis of –NH_2_-blocked CNDs. Figure S5. Representative absorption spectra of 0.02 mg/mL DPPH• methanol solution with different concentrations of the unmodified (a), –COOH-blocked (b), and –NH_2_-blocked CNDs (c) measured after reaction for 1.5 h in the dark. Figure S6. Control of the UV-Vis study of the antioxidation activity of the three types of CNDs. Figure S7. Representative cyclic voltammograms for the DPPH•-gold electrode system reacted with the unmodified CNDs at concentrations of 0.00 (a), 0.04 (b), and 0.08 mg/mL (c); –COOH-blocked CNDs at concentrations of 0.00 (d), 0.04 (e), and 0.08 mg/mL (f); and –NH_2_-blocked CNDs at concentrations of 0.00 (g), 0.04 (h), and 0.08 mg/mL (i) at scan rates of 50, 100, 150, 200, 300 and 400 mV/s, respectively. Figure S8. The antioxidation activity of the unmodified CNDs (0.02 mg/mL) in different pH values using the UV-Vis DPPH• assay. Table S1. Representative experimental results of cyclic voltammograms for the DPPH•-gold electrode system reacted with three types of CNDs at different concentrations.

## References

[1] Wang Q., Huang X., Long Y., Wang X., Zhang H., Zhu R., Liang L., Teng P., Zheng H. (2013). Hollow luminescent carbon dots for drug delivery. Carbon.

[2] Teng X., Ma C., Ge C., Yan M., Yang J., Zhang Y., Morais P.C., Bi H. (2014). Green synthesis of nitrogen-doped carbon dots from konjac flour with “off-on” fluorescence by Fe^3+^ and l-lysine for bioimaging. J. Mater. Chem. B.

[3] Albert K., Hsu H.-Y. (2016). Carbon-based materials for photo-triggered theranostic applications. Molecules.

[4] Garg B., Bisht T. (2016). Carbon nanodots as peroxidase nanozymes for biosensing. Molecules.

[5] Li H., He X., Kang Z., Huang H., Liu Y., Liu J., Lian S., Tsang Chi Him A., Yang X., Lee S.T. (2010). Water-soluble fluorescent carbon quantum dots and photocatalyst design. Angew. Chem..

[6] Wang Y., Hu A. (2014). Carbon quantum dots: Synthesis, properties and applications. J. Mater. Chem. C.

[7] Das R., Bandyopadhyay R., Pramanik P. (2018). Carbon quantum dots from natural resource: A review. Mater. Today Chem..

[8] Ray S.C., Saha A., Jana N.R., Sarkar R. (2009). Fluorescent carbon nanoparticles: Synthesis, characterization, and bioimaging application. J. Phys. Chem. C.

[9] Peng H., Travas-Sejdic J. (2009). Simple aqueous solution route to luminescent carbogenic dots from carbohydrates. Chem. Mater..

[10] Zhou J., Booker C., Li R., Zhou X., Sham T.-K., Sun X., Ding Z. (2007). An electrochemical avenue to blue luminescent nanocrystals from multiwalled carbon nanotubes (MWCNTs). J. Am. Chem. Soc..

[11] Li X., Wang H., Shimizu Y., Pyatenko A., Kawaguchi K., Koshizaki N. (2011). Preparation of carbon quantum dots with tunable photoluminescence by rapid laser passivation in ordinary organic solvents. Chem. Commun..

[12] Yang S.-T., Cao L., Luo P.G., Lu F., Wang X., Wang H., Meziani M.J., Liu Y., Qi G., Sun Y.-P. (2009). Carbon dots for optical imaging in vivo. J. Am. Chem. Soc..

[13] Yang Z.-C., Wang M., Yong A.M., Wong S.Y., Zhang X.-H., Tan H., Chang A.Y., Li X., Wang J. (2011). Intrinsically fluorescent carbon dots with tunable emission derived from hydrothermal treatment of glucose in the presence of monopotassium phosphate. Chem. Commun..

[14] Yang Y., Cui J., Zheng M., Hu C., Tan S., Xiao Y., Yang Q., Liu Y. (2012). One-step synthesis of amino-functionalized fluorescent carbon nanoparticles by hydrothermal carbonization of chitosan. Chem. Commun..

[15] Liu Y., Xiao N., Gong N., Wang H., Shi X., Gu W., Ye L. (2014). One-step microwave-assisted polyol synthesis of green luminescent carbon dots as optical nanoprobes. Carbon.

[16] Jaiswal A., Ghosh S.S., Chattopadhyay A. (2012). One step synthesis of C-dots by microwave mediated caramelization of poly(ethylene glycol). Chem. Commun..

[17] Zhai X., Zhang P., Liu C., Bai T., Li W., Dai L., Liu W. (2012). Highly luminescent carbon nanodots by microwave-assisted pyrolysis. Chem. Commun..

[18] Zhang W., Chavez J., Zeng Z., Bloom B., Sheardy A., Ji Z., Yin Z., Waldeck D.H., Jia Z., Wei J. (2018). Antioxidant Capacity of Nitrogen and Sulfur Codoped Carbon Nanodots. ACS Appl. Nano Mater..

[19] Valko M., Leibfritz D., Moncol J., Cronin M.T.D., Mazur M., Telser J. (2007). Free radicals and antioxidants in normal physiological functions and human disease. Int. J. Biochem. Cell. Biol..

[20] Jiang H., Chen F., Lagally M.G., Denes F.S. (2010). New strategy for synthesis and functionalization of carbon nanoparticles. Langmuir.

[21] Sachdev A., Gopinath P. (2015). Green synthesis of multifunctional carbon dots from coriander leaves and their potential application as antioxidants, sensors and bioimaging agents. Analyst.

[22] Zhao S., Lan M., Zhu X., Xue H., Ng T.-W., Meng X., Lee C.-S., Wang P., Zhang W. (2015). Green synthesis of bifunctional fluorescent carbon dots from garlic for cellular imaging and free radical scavenging. ACS Appl. Mater. Interfaces.

[23] Zhang W., Zeng Z., Wei J. (2017). Electrochemical study of DPPH radical scavenging for evaluating the antioxidant capacity of carbon nanodots. J. Phys. Chem. C.

[24] Paradas M., Campaña A.G., Jiménez T., Robles R., Oltra J.E., Buñuel E., Justicia J., Cárdenas D.J., Cuerva J.M. (2010). Understanding the exceptional hydrogen-atom donor characteristics of water in Ti^III^-mediated free-radical chemistry. J. Am. Chem. Soc..

[25] Hammond G.S., Boozer C.E., Hamilton C.E., Sen J.N. (1955). Air Oxidation of Hydrocarbons. III. Mechanism of Inhibitor Action in Benzene and Chlorobenzene Solutions. J. Am. Chem. Soc..

[26] Ferreira C.A., Ni D., Rosenkrans Z.T., Cai W. (2018). Scavenging of reactive oxygen and nitrogen species with nanomaterials. Nano Res..

[27] Scampicchio M., Wang J., Blasco A.J., Sanchez Arribas A., Mannino S., Escarpa A. (2006). Nanoparticle-based assays of antioxidant activity. Anal. Chem..

[28] Nie Z., Liu K.J., Zhong C.-J., Wang L.-F., Yang Y., Tian Q., Liu Y. (2007). Enhanced radical scavenging activity by antioxidant-functionalized gold nanoparticles: A novel inspiration for development of new artificial antioxidants. Free Radical Biol. Med..

[29] Pirmohamed T., Dowding J.M., Singh S., Wasserman B., Heckert E., Karakoti A.S., King J.E.S., Seal S., Self W.T. (2010). Nanoceria exhibit redox state-dependent catalase mimetic activity. Chem. Commun..

[30] Dong J., Song L., Yin J.-J., He W., Wu Y., Gu N., Zhang Y. (2014). Co_3_O_4_ nanoparticles with multi-enzyme activities and their application in immunohistochemical assay. ACS Appl. Mater. Interfaces.

[31] Zamani M., Moradi Delfani A., Jabbari M. (2018). Scavenging performance and antioxidant activity of γ-alumina nanoparticles towards DPPH free radical: Spectroscopic and DFT-D studies. Spectrochim. Acta Part A.

[32] Moura C.C., Segundo M.A., Neves J.D., Reis S., Sarmento B. (2014). Co-association of methotrexate and SPIONs into anti-CD64 antibody-conjugated PLGA nanoparticles for theranostic application. Int. J. Nanomed..

[33] Dixon H.B.F., Perham R.N. (1968). Reversible blocking of amino groups with citraconic anhydride. Biochem. J..

[34] Hill S.A., Benito-Alifonso D., Morgan D.J., Davis S.A., Berry M., Galan M.C. (2016). Three-minute synthesis of sp^3^ manocrystalline carbon dots as non-toxic fluorescent platforms for intracellular delivery. Nanoscale.

[35] Cui Q., Xu J., Wang X., Li L., Antonietti M., Shalom M. (2016). Phenyl-modified carbon nitride quantum dots with distinct photoluminescence behavior. Angew. Chem..

[36] Lim C.S., Hola K., Ambrosi A., Zboril R., Pumera M. (2015). Graphene and carbon quantum dots electrochemistry. Electrochem. Commun..

[37] Zeng Z., Zhang W., Arvapalli D.M., Bloom B., Sheardy A., Mabe T., Liu Y., Ji Z., Chevva H., Waldeck D.H. (2017). A fluorescence-electrochemical study of carbon nanodots (CNDs) in bio- and photoelectronic applications and energy gap investigation. Phys. Chem. Chem. Phys..

[38] Namdari P., Negahdari B., Eatemadi A. (2017). Synthesis, properties and biomedical applications of carbon-based quantum dots: An updated review. Biomed. Pharmacother..

[39] Duan X., Sheardown H. (2006). Dendrimer crosslinked collagen as a corneal tissue engineering scaffold: Mechanical properties and corneal epithelial cell interactions. Biomaterials.

[40] Sam S., Touahir L., Salvador Andresa J., Allongue P., Chazalviel J.-N., Gouget-Laemmel A.C., Henry de Villeneuve C., Moraillon A., Ozanam F. (2010). Semiquantitative study of the EDC/NHS activation of acid terminal groups at modified porous silicon surfaces. Langmuir.

[41] Staros J.V., Wright R.W., Swingle D.M. (1986). Enhancement by *N*-hydroxysulfosuccinimide of water-soluble carbodiimide-mediated coupling reactions. Anal. Biochem..

[42] Yang C. (2012). Enhanced physicochemical properties of collagen by using EDC/NHS-crosslinking. Bull. Mater. Sci..

[43] Kirby A.J., Lancaster P.W. (1972). Structure and efficiency in intramolecular and enzymic catalysis. Catalysis of amide hydrolysis by the carboxy-group of substituted maleamic acids. J. Chem. Soc. Perkin Trans. 2.

[44] Mostovaya O.A., Padnya P.L., Vavilova A.A., Shurpik D.N., Khairutdinov B.I., Mukhametzyanov T.A., Khannanov A.A., Kutyreva M.P., Stoikov I.I. (2018). Tetracarboxylic Acids on a Thiacalixarene Scaffold: Synthesis and Binding of Dopamine Hydrochloride. New J. Chem..

[45] Sevilla M., Fuertes A.B. (2009). Chemical and structural properties of carbonaceous products obtained by hydrothermal carbonization of saccharides. Chem. Eur. J..

[46] Solomons T.W.G., Fryhle C.B. (2011). Organic Chemistry.

[47] Bukman L., Martins A.C., Barizão É.O., Visentainer J.V., de Cinque Almeida V. (2013). DPPH Assay Adapted to the FIA System for the Determination of the Antioxidant Capacity of Wines: Optimization of the Conditions Using the Response Surface Methodology. Food Anal. Methods.

[48] Bhatti N.K., Subhani M.S., Khan A.Y., Qureshi R., Rahman A. (2006). Heterogeneous Electron Transfer Rate Constants of Viologen Monocations at a Platinum Disk Electrode. Turk. J. Chem..

[49] Muhammad H., Tahiri I.A., Muhammad M., Masood Z., Versiani M.A., Khaliq O., Latif M., Hanif M. (2016). A comprehensive heterogeneous electron transfer rate constant evaluation of dissolved oxygen in DMSO at glassy carbon electrode measured by different electrochemical methods. J. Electroanal. Chem..

[50] Hogg J.S., Lohmann D.H., Russell K.E. (1961). The kinetics of reaction of 2,2-diphenyl-1-picrylhydrazyl with phenols. Can. J. Chem..

[51] McGowan J.C., Powell T., Raw R. (1959). 630. The rates of reaction of αα-diphenyl-β-picrylhydrazyl with certain amines and phenols. J. Chem. Soc..

[52] Xie J., Schaich K.M. (2014). Re-evaluation of the 2,2-diphenyl-1-picrylhydrazyl free radical (DPPH) assay for antioxidant activity. J. Agric. Food Chem..

[53] Suzuki K., Watanabe T., Murahashi S.-I. (2013). Oxidation of primary amines to oximes with molecular oxygen using 1,1-diphenyl-2-picrylhydrazyl and WO_3_/Al_2_O_3_ as catalysts. J. Org. Chem..

[54] Galano A., Tan D.X., Reiter R.J. (2012). On the free radical scavenging activities of melatonin’s metabolites, AFMK and AMK. J. Pineal Res..

