# Supplementary material for: Tuning the Functional Groups on Carbon Nanodots and Antioxidant Studies
Source: Molecules. 2019 Jan 2;24(1):152. doi: 10.3390/molecules24010152 (PMC6337175; doi:10.3390/molecules24010152)
Supplement: Supplementary file 1 [file molecules-24-00152-s001.pdf]

Supporting Information

Tuning the Functional Groups on Carbon Nanodots and Antioxidant Studies

Zuowei Ji, Alex Sheardy, Zheng Zeng, Wendi Zhang, Harish Chevva, Kokougan Allado, Ziyu Yin, Jianjun Wei*

Department of Nanoscience, Joint School of Nanoscience and Nanoengineering, University of North Carolina at Greensboro, NC 27401, USA

*Corresponding contact:

Email: j_wei@uncg.edu, Tel: 1-336-285-2859, Fax: 1-336-500-0115

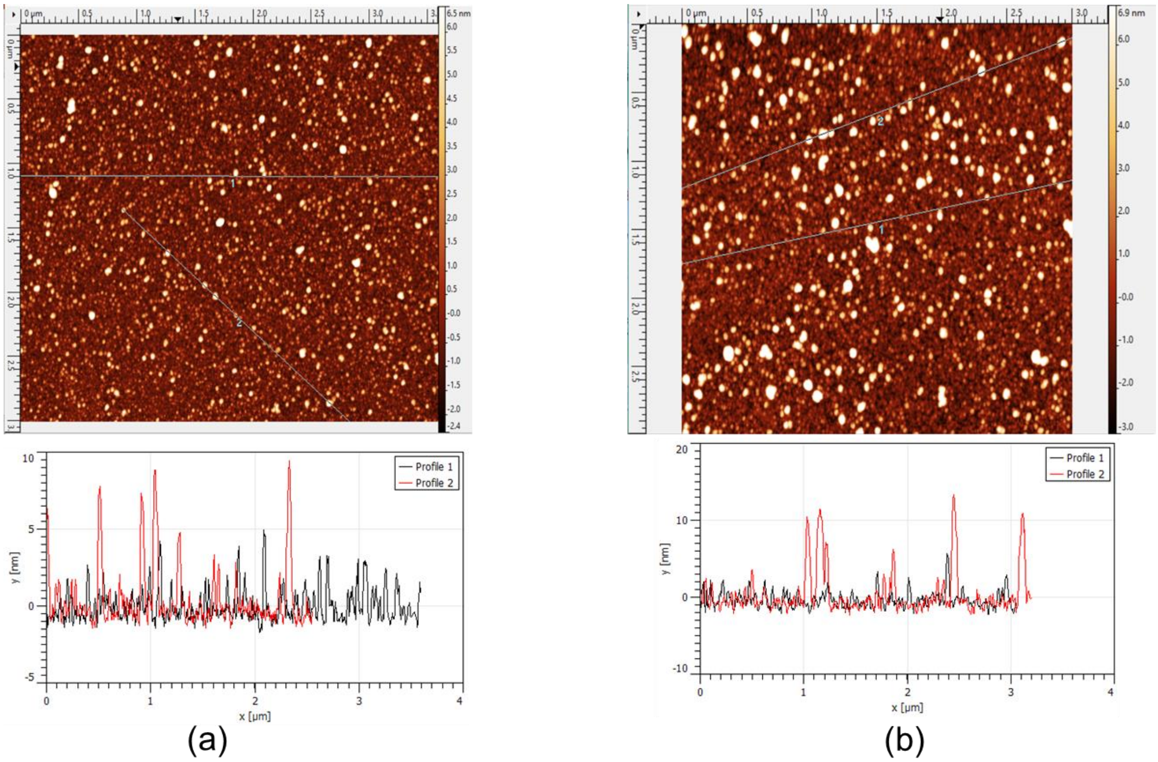

Fig. S1 AFM topography image of -COOH-blocked (a) and -NH_2-blocked CNDs (b); and the representative AFM height profiles

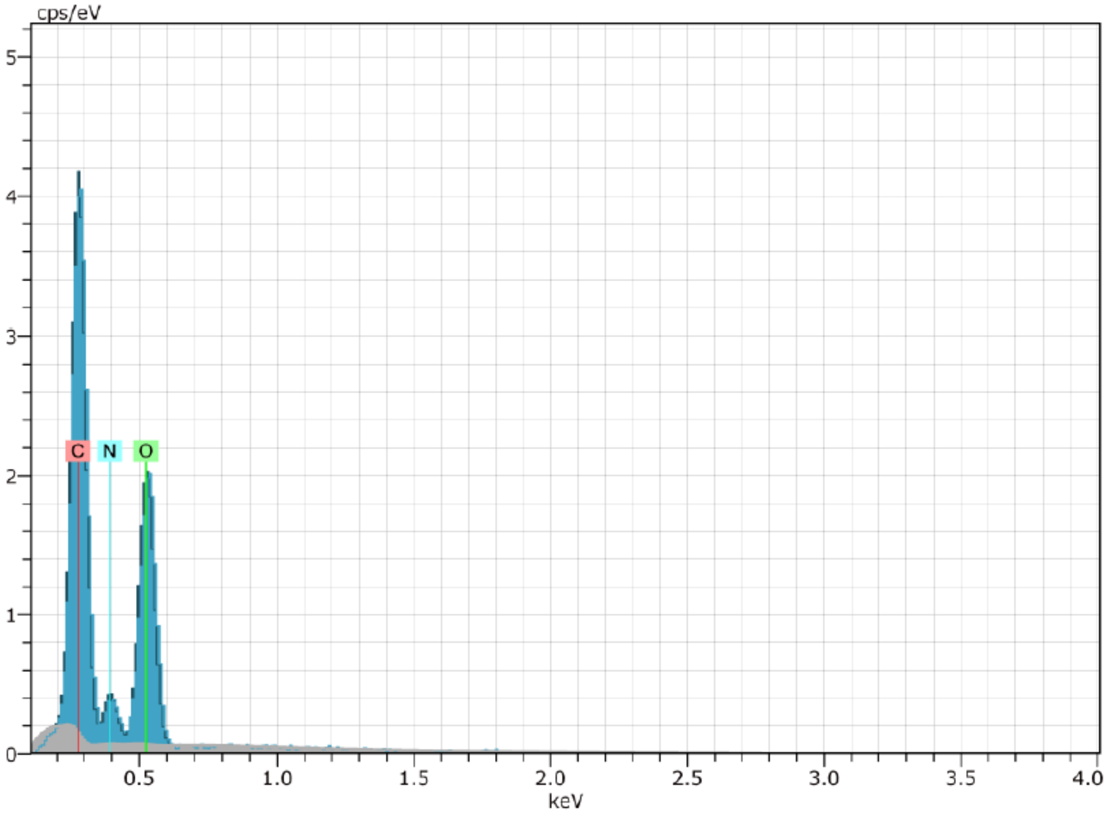


```
Bremsstrahlung    Date:11/30/2018 8:55:38 PM    HV:3.0kV    Puls th.:0.11kcps
1      Date:11/30/2018 8:55:38 PM    HV:3.0kV    Puls th.:0.63kcps
Sum    Date:11/30/2018 8:55:38 PM    HV:3.0kV    Puls th.:0.52kcps
```

Spectrum: 1

Element	Series	unn. C [wt.%]	norm. C [wt.%]	Atom. C [at.%]	Error (1 Sigma) [wt.%]
Carbon	K-series	38.19	38.19	9.92	4.98
Oxygen	K-series	28.86	28.86	5.63	4.09
Hydrogen	K-series	26.85	26.85	83.10	2.71
Nitrogen	K-series	6.09	6.09	1.36	1.23
	Total:	100.00	100.00	100.00	

WD 8.4mm
10kV
Mag 750
Apt 60um
High Current Mode
CCD Camera off
SESI detector

Fig. S2 EDX elemental analysis of un-modified CNDs

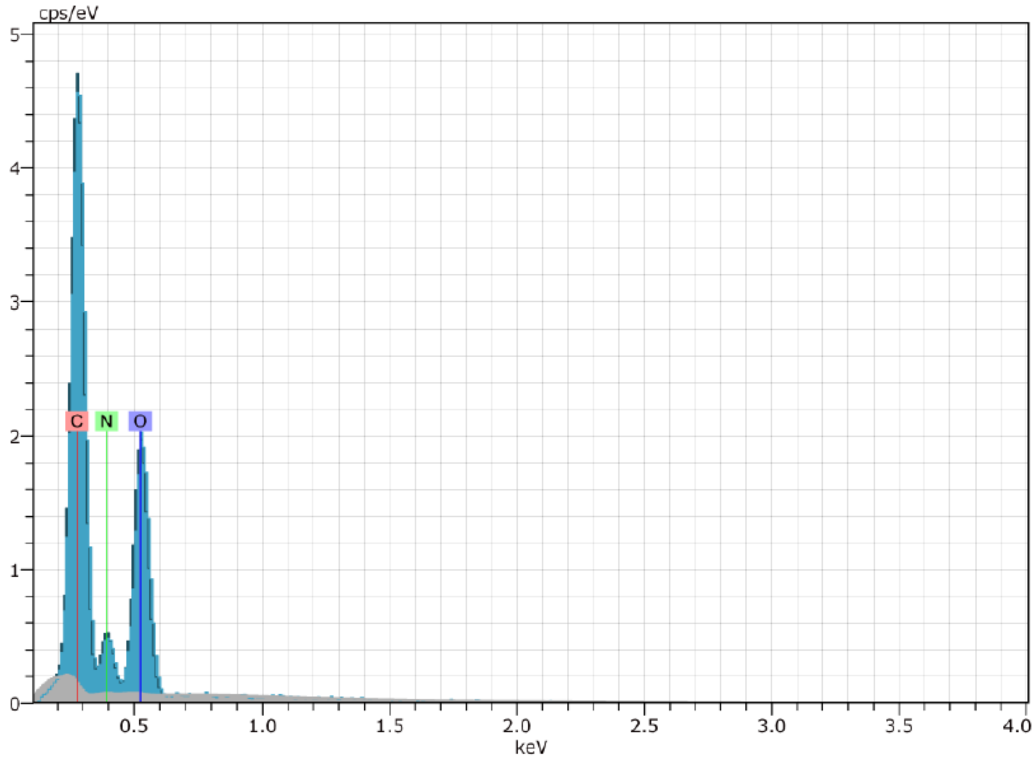

Bremsstrahlung Date:11/30/2018 9:22:46 PM HV:3.0kV Puls th.:0.11kcps
1 Date:11/30/2018 9:22:46 PM HV:3.0kV Puls th.:0.68kcps
Sum Date:11/30/2018 9:22:46 PM HV:3.0kV Puls th.:0.55kcps

Spectrum: 1

Element	Series	unn. C [wt.%]	norm. C [wt.%]	Atom. C [at.%]	Error (1 Sigma) [wt.%]
Carbon	K-series	41.46	41.46	11.73	5.34
Nitrogen	K-series	7.56	7.56	1.83	1.44
Oxygen	K-series	27.04	27.04	5.74	3.87
Hydrogen	K-series	23.94	23.94	80.69	2.42
	Total:	100.00	100.00	100.00	

WD 8.4mm
10kV
Mag 750
Apt 60um
High Current Mode
CCD Camera off
SESI detector

Fig. S3 EDX elemental analysis of -COOH-blocked CNDs

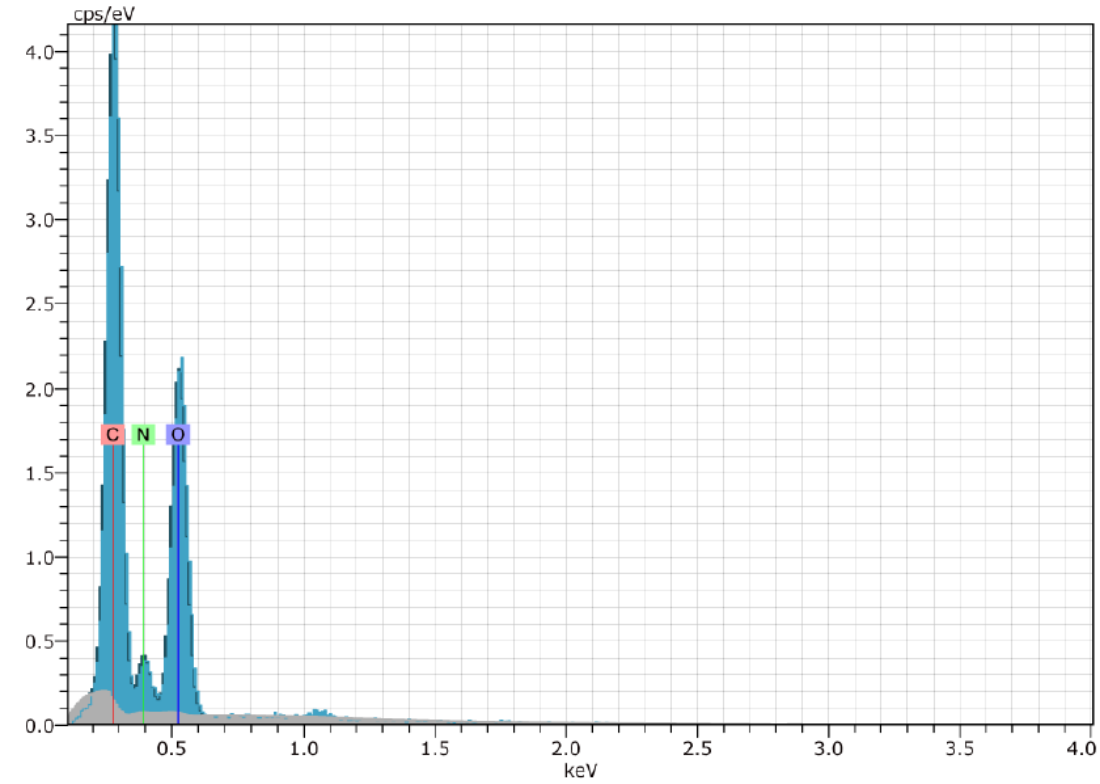

Bremsstrahlung Date:11/30/2018 9:29:57 PM HV:3.0kV Puls th.:0.11kcps
1 Date:11/30/2018 9:29:57 PM HV:3.0kV Puls th.:0.65kcps
Sum Date:11/30/2018 9:29:57 PM HV:3.0kV Puls th.:0.54kcps

Spectrum: 1

Element	Series	unn. C [wt.%]	norm. C [wt.%]	Atom. C [at.%]	Error (1 Sigma) [wt.%]
Carbon	K-series	39.29	39.29	10.90	5.09
Nitrogen	K-series	5.92	5.92	1.41	1.20
Oxygen	K-series	30.18	30.18	6.29	4.24
Hydrogen	K-series	24.62	24.62	81.40	2.49
	Total:	100.00	100.00	100.00	

WD 8.4mm
10kV
Mag 750
Apt 60um
High Current Mode

Fig. S4 EDX elemental analysis of -NH_2-blocked CNDs

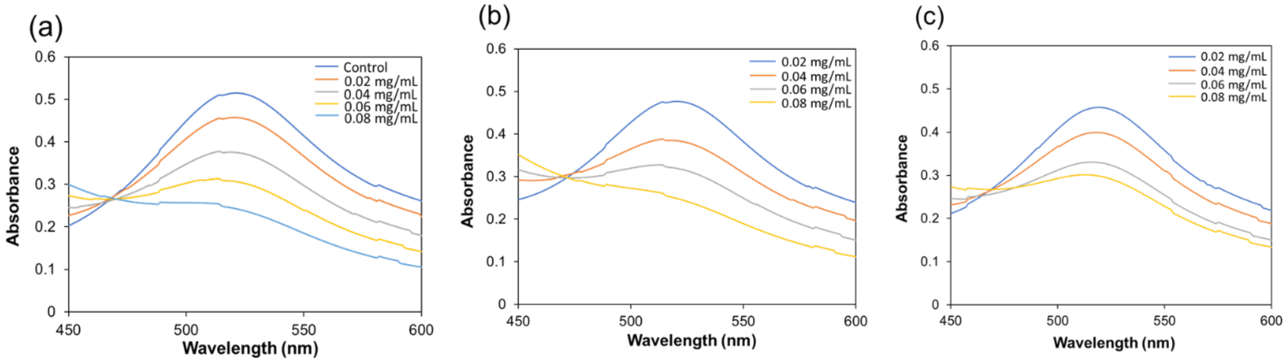

Fig. S5 Representative absorption spectra of 0.02 mg/mL DPPH• methanol solution with different concentrations of the unmodified (a), -COOH-blocked (b), and $-NH_2$-blocked CNDs (c) measured after reaction for 1.5 hours in the dark.

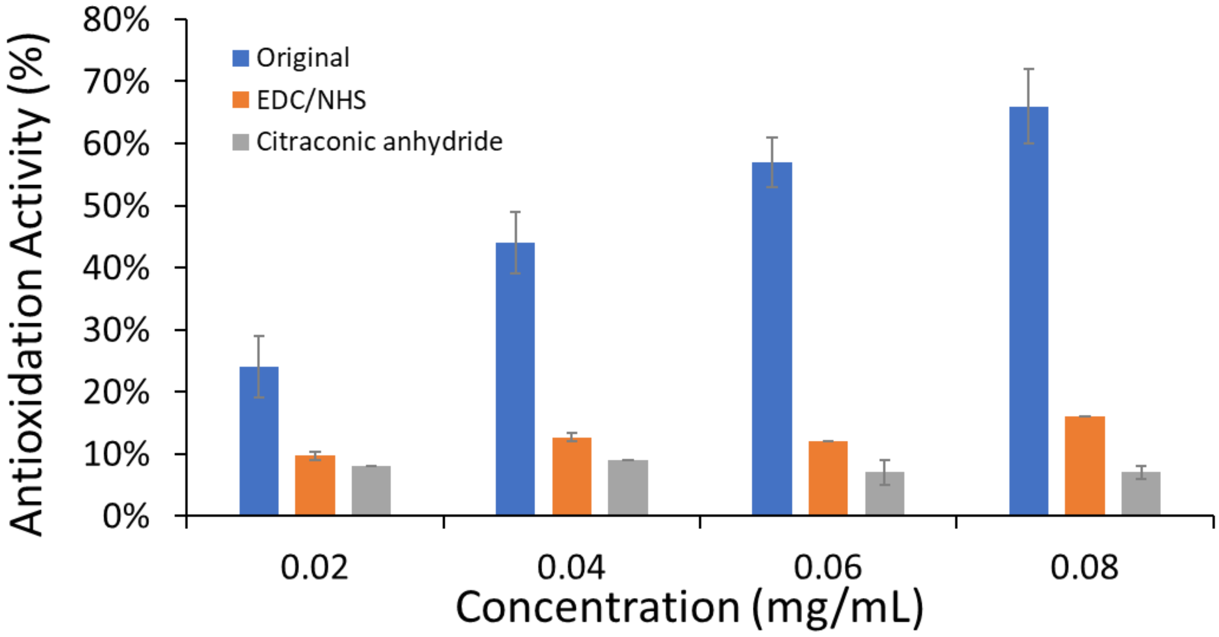

Fig. S6 Control of the UV-Vis study of the antioxidation activity of the three types of CNDs.

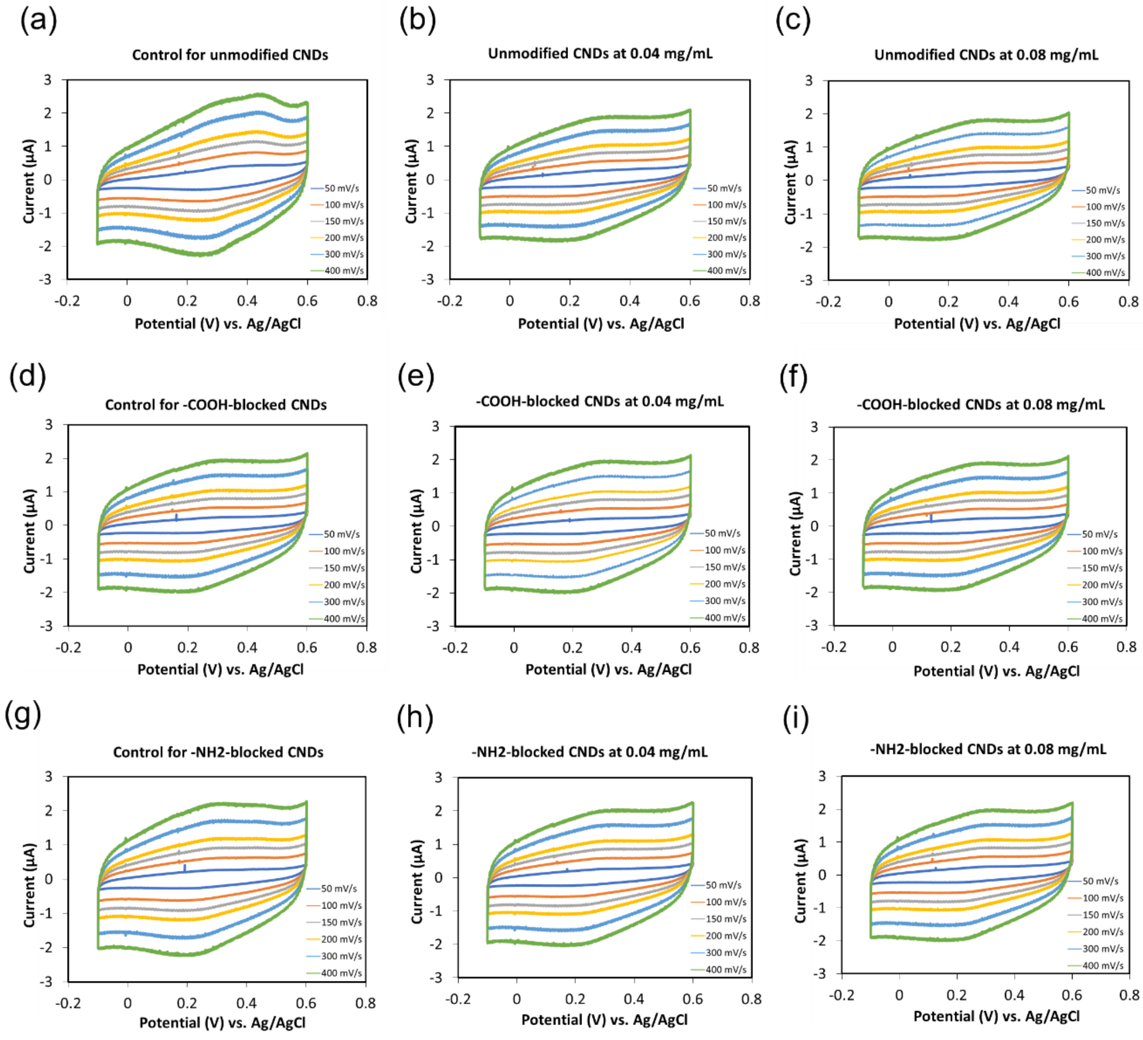

Fig. S7 Representative cyclic voltammograms for the DPPH•-gold electrode system reacted with the unmodified CNDs at concentrations of 0.00 (a), 0.04 (b), and 0.08 mg/mL (c); -COOH-blocked CNDs at concentrations of 0.00 (d), 0.04 (e), and 0.08 mg/mL (f); and -NH_2-blocked CNDs at concentrations of 0.00 (g), 0.04 (h), and 0.08 mg/mL (i) at scan rates of 50, 100, 150, 200, 300 and 400 mV/s, respectively.

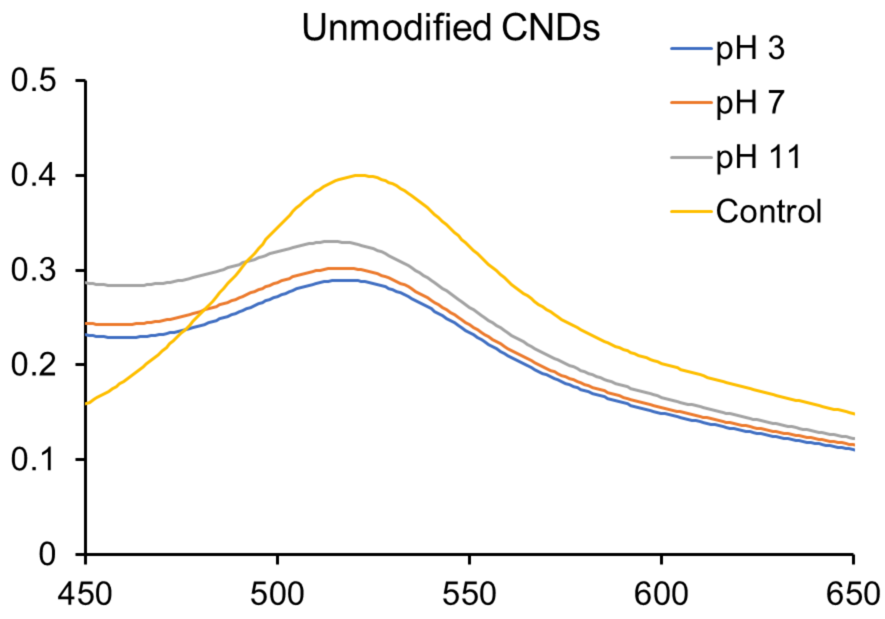

Antioxidation activity	pH =3	pH =7	pH =11
Unmidified CNDs	27%	23%	17%

Fig. S8. The antioxidation activity of the unmodified CNDs (0.02 mg/mL) in different pH values using the UV-Vis DPPH• assay.

Table S1. Representative experimental results of cyclic voltammograms for the DPPH•-gold electrode system reacted with three types of CNDs at different concentrations.

Concentration of CNDs (mg/mL)		Unmodified CNDs	-COOH-blocked CNDs	-NH2-blocked CNDs
0.00	Initial $C_{DPPH•}$ (nmol/mL)	50.8	50.8	50.8
	Slope of dependence	2.98	1.94	2.64
	D_0 (cm^2/s)	$2.58*10^{-5}$	$1.08*10^{-5}$	$2.01*10^{-5}$
0.04	Slope of dependence	1.94	1.91	2.13
	Reserved $C_{DPPH•}$ (nmol/mL)	33.1	50.1	41.1
0.08	Slope of dependence	1.81	1.80	2.07
	Reserved $C_{DPPH•}$ (nmol/mL)	30.8	47.2	39.9
